# Early Implementation of a Nationwide Opt-Out Electronic Health Record for Outpatient Care in Germany: Qualitative Semistructured Interview Study

**DOI:** 10.2196/94620

**Published:** 2026-09-21

**Authors:** Susann May, Felix Muehlensiepen, Gina Barzen, Frances Seifert, Manuela Marquardt, Liane Schenk, Gerhard Hindricks, Sebastian Spethmann

**Affiliations:** 1 German Heart Center at Charité – University Medical Center Berlin, corporate member of Freie Universität Berlin and Humboldt-Universität zu Berlin, Department of Cardiology, Angiology and Intensive Care Medicine, Chariteplatz 1, Berlin, 10117, Germany, +49 3045050; 2 User Experience in Digital Health Lab, Center for Health Services Research Brandenburg, Faculty of Health Sciences Brandenburg, Brandenburg Medical School Theodor Fontane, Rüdersdorf/Neuruppin, Germany; 3 Deutsches Zentrum für Herzkreislaufforschung (DZHK), Berlin, Germany; 4 Charité – University Medical Center Berlin, corporate member of Freie Universität Berlin and Humboldt-Universität zu Berlin, Institute of Medical Sociology and Rehabilitation Science, Berlin, Germany

**Keywords:** electronic health record, qualitative analysis, digital health, Consolidated Framework of Implementation Research, implementation research, patient empowerment

## Abstract

**Background:**

Electronic health records are a central component of digital health strategies worldwide. Early implementation phases are particularly critical for shaping long-term adoption patterns but remain insufficiently studied, especially in large-scale, policy-driven rollouts. In January 2025, Germany introduced the electronic patient record (*elektronische Patientenakte*, ePA) as a nationwide opt-out system, creating a unique opportunity to examine early implementation in routine outpatient care.

**Objective:**

This study aimed to explore facilitators and barriers influencing the early implementation of the ePA in German outpatient practices from the perspective of health care professionals.

**Methods:**

A qualitative study based on semistructured interviews with general practitioners, medical assistants, and physician assistants was conducted between August and December 2025. Participants were recruited nationwide through regional physician associations and a professional organization using a criterion-based purposive recruitment strategy with maximum variation elements. Interviews were analyzed using qualitative content analysis guided by the Consolidated Framework for Implementation Research. Coding combined deductive framework-based and inductive thematic approaches.

**Results:**

A total of 49 interviews were conducted. Facilitators and barriers were identified across all 5 Consolidated Framework for Implementation Research domains, with the largest number of categories in the inner setting. Organizational factors such as leadership engagement, internal communication structures, and learning climate were central facilitators of implementation. In contrast, barriers were primarily related to technical instability, heterogeneous functionality of practice management systems, limited interoperability across care sectors, and insufficient patient awareness. Although the ePA was generally perceived as intuitive and potentially beneficial, particularly for improving information availability and reducing redundant diagnostics, its routine use remained strongly dependent on organizational capacity, infrastructure stability, and cross-sectoral integration.

**Conclusions:**

Early implementation of the nationwide opt-out ePA in Germany is shaped less by professional resistance than by organizational, technical, and systemic conditions. These findings highlight that successful large-scale digital health implementation requires coordinated strategies addressing technical infrastructure, organizational readiness, governance, and patient engagement beyond the mere deployment of digital systems.

## Introduction

The widespread adoption of electronic health records (EHRs) is a key component of digital health strategies around the world. The expectation is that EHRs will enhance continuity of information, reduce unnecessary diagnostics, and strengthen patient engagement [1]. However, international experience demonstrates that technical availability alone does not guarantee sustained, meaningful use in routine care. EHR implementation is further shaped by factors such as data governance, privacy concerns, and public trust, which influence both professional and patient engagement with digital health systems [2-4]. Accordingly, EHRs can be considered complex sociotechnical interventions, the implementation of which is influenced by the interaction of technical, organizational, professional, and systemic conditions [5,6]. Despite extensive research on the adoption and usability of EHRs, empirical evidence on early implementation, particularly in large-scale rollouts, remains limited, with existing studies often providing descriptive insights into implementation processes [7,8].

In this regard, there is a paucity of research concerning the manner in which health care professionals (HCPs) assimilate recently mandated EHRs into their routine clinical workflows, particularly in instances where implementation is driven by policy decisions as opposed to local initiatives. This is especially relevant for nationwide, policy-driven implementations in which adoption is not voluntary but structurally embedded within health care systems. The early implementation phase is of critical importance, as it has been demonstrated to shape long-term usage patterns, organizational routines, and professional engagement. However, as yet, it remains underrepresented in implementation research. In particular, little is known about how HCPs integrate mandated EHR systems into routine care during the early rollout phase in real-world settings beyond pilot or controlled environments.

Germany offers a distinctive and informative case study for examining the early implementation of EHRs at a national scale. Germany’s health care system is characterized by a high degree of fragmentation, strong self-governance [9], and comparatively low levels of digitalization, lagging behind international peers and posing specific challenges for large-scale digital health implementation [10]. In January 2025, the German electronic patient record (*elektronische Patientenakte*, ePA) was introduced as a nationwide opt-out system [11], making EHRs available to nearly all individuals with statutory health insurance. This reform transformed a previously modest opt-in system [12] into a policy-driven, population-wide intervention, constituting a natural experiment in large-scale digital health implementation within a fragmented health care system. As such, the ePA rollout provides a unique opportunity to study how a nationwide, policy-driven digital health intervention is implemented and integrated into routine outpatient care.

The present study examines the early implementation of the ePA in German outpatient care from the perspective of HCPs. We used the Consolidated Framework for Implementation Research (CFIR) to identify determinants across the innovation, inner setting, outer setting, individual, and implementation process domains [13]. This multilevel perspective is particularly suited to examining a nationwide, policy-driven intervention whose implementation depends on the interplay of technological, organizational, professional, and system-level conditions. In contrast, the Technology Acceptance Model explains technology adoption primarily through perceived usefulness and perceived ease of use [14] and therefore captures the broader implementation context only partially. We addressed the following research question: Which multilevel factors across CFIR domains influence the early implementation and integration of the ePA into routine outpatient care from the perspective of HCPs?

By examining routine care rather than a pilot setting, the study provides theory-informed insights into why nationwide EHR availability does not necessarily translate into successful implementation.

## Methods

### Study Design

This qualitative interview study was conducted as part of ePA4all, a mixed methods project examining the implementation of the ePA in Germany from multiple stakeholder perspectives [15]. The present study focused on how general practitioners, medical assistants, and physician assistants experienced the early implementation of the ePA in routine outpatient care. We used the updated CFIR to identify multilevel implementation determinants. Reporting followed the COREQ (Consolidated Criteria for Reporting Qualitative Research; Checklist 1) [16].

### Intervention

**Figure 1.**
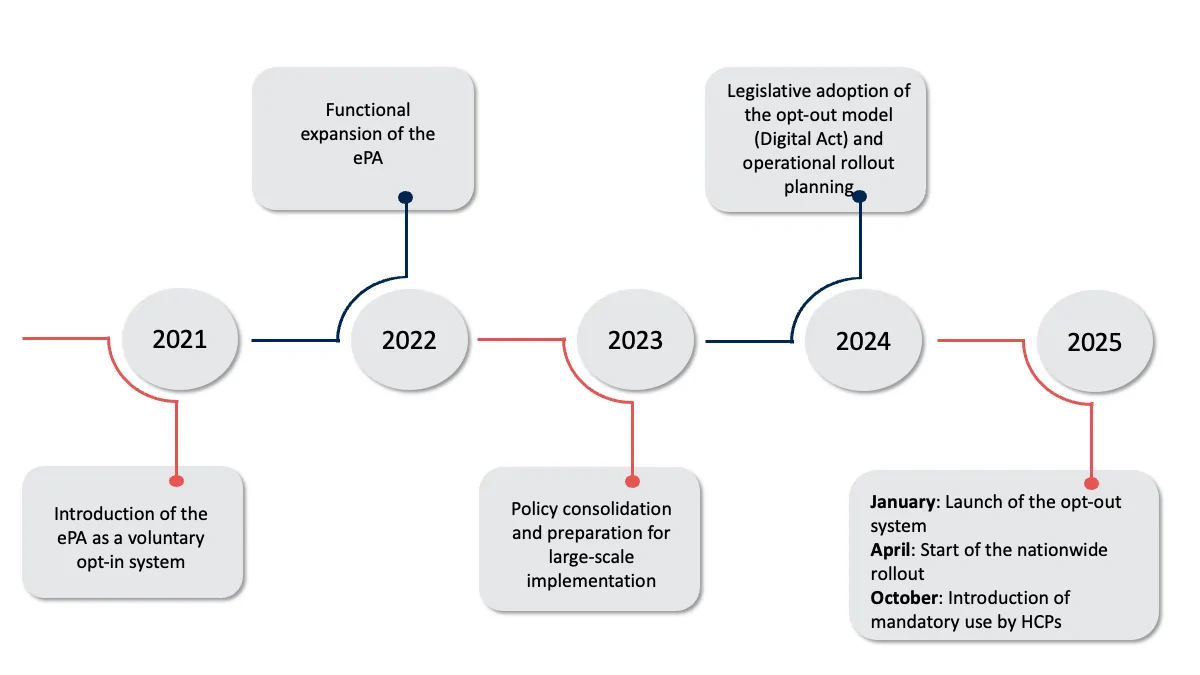
Timeline of milestones in the implementation of the *elektronische Patientenakte* (German electronic health record) (ePA) in Germany. HCPs: health care professionals.

The intervention under study was the nationwide introduction and early rollout of the German ePA as a policy-driven digital health intervention. The ePA is a patient-managed digital health record designed to store and share health-related information, such as medical reports, diagnostic findings, and medication data, across health care sectors. It is accessible to patients and authorized HCPs and is intended to support information continuity and reduce redundant examinations. Strategic governance is led by the Federal Ministry of Health, while the national digital health agency gematik is responsible for technical development and operational coordination. Health insurers are legally required to provide an ePA to their insured members, whereas health care providers are responsible for its use in routine care. The ePA was initially introduced in January 2021 as a voluntary opt-in system but achieved limited uptake. In response, the Digital Act [11] initiated a strategic shift toward an opt-out model. Since January 2025, statutory health insurers have been required to provide an ePA for all insured individuals unless they actively object. Implementation followed a staged approach, including regional pilot testing in selected regions, a gradual nationwide rollout starting in April 2025, and mandatory usage by health care providers from October 2025 onward (Figure 1). During the study period, the ePA was technically available in participating general practices and could be accessed via existing practice management systems (PMSs).

HCPs were able to view the ePA within their routine software environment and to upload and retrieve clinical documents, such as physician reports and discharge summaries, as well as access medication information. Importantly, the rollout did not include additional study-specific implementation support; use of the ePA occurred under routine care conditions shaped by existing organizational, technical, and policy frameworks.

### Ethical Considerations

All study procedures were approved by the Ethics Committee of Charité-Universitätsmedizin Berlin (reference ID: EA1/333/24). The study was conducted in accordance with relevant ethical guidelines and regulations. Participants were informed verbally and in writing about the purpose, procedure, and significance of the study, as well as the associated benefits and risks, and were given the opportunity to ask questions. They were also informed that they had the right to withdraw their consent to participate in the study at any time, either verbally or in writing, without giving reasons. They were also informed that personal data would be collected and stored, whereby the data would be anonymized, but no data would be published that could be used to identify the individual. For security reasons, the data received from participants were always stored in a password-protected folder on a secure desktop computer. Written consent was obtained after participants had the opportunity to ask questions. Patients were not involved in the design of this study. Participants were offered €50 (approximately US $59.48) as an incentive for their participation in the study.

### Setting

The study was conducted in the German outpatient setting and focused on the early implementation of the ePA. Data were collected nationwide in outpatient practice settings, including primary care practices of varying sizes, ranging from small single-physician practices to larger group practices. The sample comprised both regular practices and selected pilot practices that had been involved in early testing phases of the ePA. Experiences from the early rollout phase were not used as outcome data to select practices; instead, recruitment aimed to include participants with initial practical exposure to the ePA across different practice types and regions. Data collection spanned a transitional period in the ePA rollout. Between August and September 2025, the ePA was nationally available, but its use by HCPs was not yet mandatory. From October 2025 onward, mandatory use had begun, although the extent of integration into routine clinical workflows continued to vary across practices.

### Participants

Participants were recruited with the support of regional Associations of Statutory Health Insurance Physicians (Kassenärztliche Vereinigungen) in Germany, including the Associations in Westphalia-Lippe (KVWL), Schleswig-Holstein (KVSH), Lower Saxony (KVN), and Berlin (KVB), as well as the German Association for Physician Assistants (Deutsche Gesellschaft für Physician Assistants). The Associations of Statutory Health Insurance Physicians contacted general practitioners and medical assistants in outpatient general practice settings within their respective regions and distributed general study invitations. In parallel, physician assistants were recruited by the German Association for Physician Assistants through a study announcement published on its website and via direct email communication to its members. The organizations did not identify or individually target potential participants. Interested individuals contacted the study team in response to these invitations and were subsequently assessed for eligibility.

Participants were selected using a criterion-based purposive recruitment strategy with maximum variation elements [17]. Eligible participants were adults (≥18 y) working as general practitioners, medical assistants, or physician assistants in outpatient general practice, with sufficient German language proficiency and initial experience with the ePA. Nurse practitioners were not included because this professional role is not routinely established as part of standard outpatient general practice teams in Germany. Such teams typically comprise general practitioners and medical assistants, while physician assistants are increasingly involved in some practices. Nurse practitioners were therefore not part of the predefined target population. Prior ePA experience was required to ensure that participants could report on concrete implementation experiences rather than hypothetical expectations. No minimum number of years of professional experience or predefined level of digital technology use was specified as an eligibility criterion because the study aimed to capture a broad range of perspectives on early ePA implementation. Restricting eligibility based on these characteristics could have excluded relevant implementation experiences. Professional experience and technical affinity were therefore recorded descriptively to characterize the sample rather than used to determine eligibility. Sampling aimed to achieve variation across professional roles, regions, and practice settings. All participants provided informed consent prior to participation.

### Data Collection

Data were collected between August and December 2025 during the early rollout phase of the opt-out ePA using semistructured qualitative interviews. No interview data from the previous voluntary opt-in phase were included in this analysis. An interview guide was developed by a multiprofessional research team and was informed by the study objectives and CFIR [13]. The interview guide comprised open-ended questions aimed at exploring participants’ experiences with the ePA, perceived benefits and challenges of its use in routine care, integration into existing workflows, and contextual factors influencing implementation at the practice level.

The interview guide was pilot-tested with 5 eligible participants to assess the clarity, comprehensibility, relevance, and flow of the questions. Pilot participants completed the interview and provided feedback on wording and content. As only minor wording changes were made and no substantial changes to the interview guide were required, the pilot interviews were included in the final analysis. No specific usage requirements or implementation strategies were introduced as part of the study. Participants were asked to reflect on their real-world experiences with the ePA under routine conditions (please see the interview guide in Multimedia Appendix 1). Additionally, sociodemographic and professional characteristics of the participants were collected, including age, sex, professional role, years of professional experience, and the population size of the practice location; technical affinity was further assessed using a standardized scale (Affinity for Technology Interaction Short Scale) [18].

All interviews were conducted remotely via telephone (SM and FS). Telephone interviews were chosen to facilitate nationwide recruitment and flexible participation among HCPs working in busy outpatient settings. This format minimized organizational and technical barriers, did not require participants to use additional videoconferencing software, and was considered appropriate because the study focused on verbal accounts of implementation experiences rather than on visual interaction or observation. Interviews were audio-recorded with participants’ consent and transcribed verbatim in accordance with data protection regulations. In addition, brief field notes were documented after each interview to capture contextual impressions and support interpretation of the interview situation; these notes were not included in the formal analysis. Data collection continued until both code saturation, defined as the absence of additional issues, and meaning saturation, defined as the absence of further dimensions, nuances, or insights, were reached across the overall sample [19]. Saturation was not assessed separately for each professional group because the study aimed to identify multilevel implementation determinants across outpatient practice teams rather than compare professional groups.

### Data Analysis

Data analysis followed a qualitative, theory-informed approach guided by the CFIR. Interview transcripts were analyzed using qualitative content analysis according to Kuckartz, combining deductive and inductive coding [20]. Deductive coding was used to structure the analysis according to the updated CFIR domains and constructs. Within these deductively applied constructs, inductive coding was used to develop data-driven categories that reflected specific implementation determinants emerging from the interview material. These inductive categories were iteratively refined during coding and team discussions and were then assigned to the CFIR construct that best represented their primary analytical meaning. The analysis was conducted using MAXQDA Analytics Pro 2022 (Release 22.1.0; VERBI Software GmbH) by 2 members of the study team (SM and FM). Both analysts participated in the coding process and regularly discussed coding decisions, category definitions, and ambiguous segments. Discrepancies were resolved by consensus, involving the broader research team when necessary. No formal intercoder reliability coefficient was calculated; instead, an iterative consensus-based approach was chosen to support the collaborative refinement and consistent application of the coding framework.

Coding was conducted iteratively and in parallel with data collection to allow for continuous refinement of the coding scheme. No member checking was conducted. All interviews were conducted and analyzed in German. For reporting purposes, illustrative quotations were selected and translated into English; translations were reviewed within the research team to ensure semantic accuracy and preserve contextual meaning. Qualitative frequency descriptors, including “a few,” “some,” “several,” and “many,” were used to provide a transparent indication of the relative distribution of findings within the dataset. These terms are descriptive rather than statistical because the semistructured interviews did not elicit every determinant systematically from every participant; accordingly, they should not be interpreted as prevalence estimates.

## Results

### Participant Characteristics

In total, 49 interviews were conducted and analyzed. The mean duration of the interviews was 42 (SD 12.7; range 23‐70) minutes. The mean age of the participants was 43 (SD 11.0; range 22‐63) years. Most participants were female (35 female/14 male). A total of 20 general practitioners, 20 medical assistants, and 9 physician assistants participated. A total of 5 pilot practices took part. Detailed characteristics of study participants are shown in Table 1.

**Table 1.** Sample characteristics and affinity for technology interaction (ATI) score (computed as the mean of ATI interaction items; scale range 1‐6; categorized as low 1.00‐2.66, moderate 2.67‐4.32, high 4.33‐6.00).

Characteristics	Participants (N=49), n (%)
Age (y)	
<30	8 (16.3)
30‐40	10 (20.4)
41‐50	19 (38.8)
51‐60	8 (16.3)
61‐70	4 (8.2)
Sex	
Female	35 (71.4)
Male	14 (28.6)
Nonbinary	0 (0)
Duration of professional activity (y)	
<10	18 (36.7)
10‐20	19 (38.8)
21‐30	7 (14.3)
31‐40	4 (8.2)
>40	1 (2.0)
Missing	0 (0)
Population size of practice location (number of inhabitants)	
<5000	2 (4.1)
5001‐20,000	12 (24.5)
20,001‐100,000	17 (34.7)
100,001‐1 million	9 (18.4)
>1 million	9 (18.4)
Profession	
General practitioner	20 (40.8)
Medical assistant	20 (40.8)
Physician assistant	9 (18.4)
Technical affinity	
High	25 (52.1)
Moderate	19 (39.6)
Low	4 (8.3)
Missing	1 (2.0)

The analysis identified 47 data-driven categories across all 5 CFIR domains. The Innovation domain comprised 8 facilitators and 1 barrier, the Outer Setting 1 facilitator and 3 barriers, the Inner Setting 8 facilitators and 11 barriers, the Individuals domain 5 facilitators and 5 barriers, and the Implementation Process 1 facilitator and 4 barriers. Table 2 presents shortened labels for these categories to provide a concise overview. The complete coding tree, including the full category labels, corresponding updated CFIR constructs, and anchor quotations, is provided in Multimedia Appendix 2.

**Table 2.** Overview of facilitators and barriers influencing early *elektronische Patientenakte* (German electronic health record) implementation across Consolidated Framework for Implementation Research domains.

CFIR^a^ domain	Facilitators	Barriers
Innovation	Intuitive usability Protected trial phase Workflow optimization during trial Visible refinement following feedback Increasing usefulness Fewer redundant examinations Improved information availability Greater patient transparency and engagement	Complexity caused by technical instability
Outer Setting	Financial incentives as initial trigger	Limited vendor accountability Sectoral implementation gaps Insufficient patient information from health insurers
Inner Setting	Low-threshold knowledge resources Learning by doing Structured internal exchange Workflow-neutral integration Continuous team communication Dedicated digital roles Shared need for change Digitally oriented practice culture	Insufficient practice-oriented training Legal and data-protection uncertainty Limited IT infrastructure Staffing and time constraints Investment costs and technical dependencies Additional counseling and support burden Parallel documentation systems PMS^b^-dependent compatibility Incompatible data formats Interference with other digital applications Limited structural flexibility
Individuals: Roles and Characteristics	Active leadership Positive appraisal of digital work Technical affinity and prior experience Readiness for change and initiative Personal commitment	Low patient demand and perceived relevance Patient knowledge deficits Authentication and registration barriers Digital fatigue and frustration Preference for paper-based work
Implementation Process	Increasing habituation and routinization	Informal planning Fragmented workflow integration Additional initial workload Heterogeneous routine use

a CFIR: Consolidated Framework for Implementation Research.

b PMS: practice management system.

### Innovation

The Innovation domain captured how participants perceived the ePA itself, including its complexity, trialability, adaptability, and relative advantage. Overall, the ePA was often described as easy to access and intuitive when the surrounding technical environment functioned reliably. Perceived intuitive usability was reflected in descriptions of the ePA as straightforward and requiring only a few steps.

*It’s really, really completely straightforward. It’s genuinely simple. It’s literally one click and the ePA opens immediately.*

[GP16_male_49]

However, perceived complexity increased when technical disruptions or system instability occurred. In these situations, the ePA was not experienced as conceptually difficult, but as unreliable and time-consuming in routine practice.

*Sometimes it doesn’t work, which still happens occasionally. Then you’re sitting there and don’t know why, and it ends up taking five minutes.*

[GP12_female_52]

Trialability was described as an important facilitator of early implementation. Some participants valued a protected trial phase without immediate implementation pressure because it created space for familiarization, error detection, and adjustment before mandatory use. The trial phase was also used to test and optimize workflows, enabling practices to identify practical problems and address them together with IT service providers.

*Before it became mandatory, it was important to first try out the workflow in practice, in order to identify problems early on and discuss and resolve them with our IT service provider.*

[GP14_male_61]

Adaptability was mainly experienced through visible system modifications following user feedback. Such changes increased the perception that feedback from practices was taken seriously and that the system could be refined in response to practical needs.

*After a few weeks or months, you can actually see that your feedback has been heard, for example, when a button is no longer in the top right corner but has been moved to the bottom left, where it is easier to reach.*

[GP5_male_54]

Many participants perceived relative advantages of the ePA, whose benefits were expected to increase as use continued and more information became available.

*For us, the ePA is genuinely useful, very useful, and it becomes more useful every day. It’s not hindering my work in any way.*

[GP5_male_54]

Expected benefits included reduced redundant examinations and improved information availability, particularly when data from other providers were accessible across sectors.

*...that at some point this will no longer be necessary, because every specialist takes their own blood samples anyway. So that such redundant examinations can simply be avoided.*

[GP8_female_42]

The ePA was seen as a repository that could compensate for incomplete patient documentation or missing paper-based information.

*The patient has everything with them, because they often don’t bring their medication plan or their reports, you know.*

[MA4_female_33]

Another perceived advantage was increased transparency for patients. Several participants described the ePA as a tool that could strengthen patient overview and engagement with their own health information.

### Outer Setting

The Outer Setting comprised findings related to the CFIR constructs of financing, policies and laws, and partnerships and connections. Financial incentives were described as an initial trigger for implementation, particularly regarding reimbursable initial data entry. However, several participants also indicated that such incentives were limited in their ability to support sustained integration into routine workflows.

*At the moment, the main excitement is really about rushing to claim the initial data entry fee before it gets abolished again (laughs). Many colleagues just want to collect the eleven euros.*

[GP14_male_61]

A major external barrier concerned the perceived lack of binding requirements and enforcement mechanisms for PMS manufacturers. Participants attributed heterogeneous functionality and usability across PMS to insufficient accountability at the vendor level. Sectoral implementation gaps further limited the perceived usefulness of the ePA. Many participants reported that their own efforts within general practices were undermined when other care sectors were not able or willing to access and use the ePA.

*I do this with my patients as well. I adapt things [in the ePA] when I change something myself. But it’s all pointless if they end up in hospital and there’s no one there who can even open an electronic patient record.*

[GP13_male_55]

Several participants reported that health insurance companies did not adequately inform patients about the ePA, thereby shifting additional explanatory work to outpatient practices.

### Inner Setting

Within the Inner Setting, findings were organized according to CFIR constructs including access to knowledge and information, available resources, compatibility, relational connections, communications, culture, and tension for change. The Inner Setting contained the largest number of implementation determinants. Access to knowledge and information was a central condition of implementation readiness. Structured, low-threshold knowledge resources provided by PMS manufacturers, such as e-learning platforms and short videos, were described as helpful. At the same time, several participants reported insufficient, nonpractice-oriented, or absent training opportunities. Several participants expressed concerns about the management of sensitive or potentially stigmatizing information, patient control over information, and the need for clearer guidance.

*I don’t deny that the patient record can make sense in general. But for people with stigmatizing conditions, who don’t want others to know that they take HIV medication or opioids for addiction, it is communicated far too little how they can prevent this.*

[GP3_male_58]

Available resources were understood as practical operational capacity. Technical infrastructure limitations, including unstable connections and insufficient system capacity, were described as barriers to low-threshold use. Limited staffing resources and persistent time constraints further restricted implementation. In high-volume practices, additional ePA-related tasks competed with already dense routine workflows.

*We simply don’t have the time to think about uploading data to the ePA, because there are already four other patients waiting.*

[PA2_female_27]

Several participants also linked implementation to investment costs and technical dependencies, which were experienced as additional workload and financial burden.

*When you take all of this into account, the workload is enormous and costly.*

[MA4_female_33]

The learning climate in practices was characterized by informal and practice-based learning. Learning by doing was common, especially after initial formal information or training had ended.

*After that, everything was basically over, and it became a matter of learning by doing.*

[GP5_male_54]

Established formats for internal exchange and reflection, such as quality circles or regular team meetings, supported collective learning and problem-solving.

*We have a quality circle in our practice, where there is a lot of exchange.*

[GP12_female_52]

Compatibility with existing workflows was assessed heterogeneously. A few participants reported workflow-neutral integration when ePA use did not require changes to established routines. In other cases, parallel documentation systems led to media discontinuities and additional manual steps. These discontinuities made routine use difficult to sustain.

*Then I would have to download it each time, save it, and transfer it into [name of PMS]. Of course, we can’t manage that.*

[MA4_female_33]

Compatibility was strongly dependent on the respective PMS. Several participants compared their own systems with those used by colleagues and reported substantial differences in upload functions and workflow fit.

*A friend of mine uses a different system, and for them it works relatively smoothly. They can upload documents easily and even upload several at once. We always have to upload them individually.*

[GP14_male_61]

Incompatible data formats and complex conversion processes were perceived as particularly burdensome. Many participants described multistep conversion procedures as a major barrier to uploading self-generated findings. Some participants also described negative interactions with other digital applications, indicating that the ePA was embedded in a fragile broader digital infrastructure. Networks and communication within the practice were important facilitators. Continuous internal communication and team feedback helped identify problems and adapt implementation processes. Structural characteristics shaped the extent to which implementation could be organized. Clearly embedded roles, such as a digital manager, facilitated coordination and communication within the team. By contrast, larger organizations were sometimes described as having limited structural flexibility, making it difficult to create dedicated digital roles or respond quickly to implementation needs.

*We simply don’t have the capacity to employ someone like that. We are light-years away from being able to do so.*

[PA2_female_27]

Culture and tension for change influenced whether digitalization was treated as a collective task. In some practices, a few participants described a shared sense that change required joint effort. A positively framed, digitally oriented practice culture facilitated ePA implementation by normalizing digital work and encouraging active contributions from the team.

*We work completely digitally, and the nursing staff are fully on board. We try to implement this wherever possible and also contribute ideas on how processes could be further digitalized.*

[GP8_female_42]

### Individuals: Roles and Characteristics

Within the Individuals domain, patients were conceptualized according to the CFIR role of innovation recipients, while HCP-related findings were examined through relevant individual roles and characteristics. Many participants described low patient demand and limited perceived relevance of the ePA in everyday consultations.

*The ePA hardly ever comes up in consultations with patients. Patients ask about it very rarely.*

[GP5_male_54]

Many participants linked low patient demand to limited knowledge about the opt-out regulation, the content of the ePA, and the actions required from patients.

*Patients often asked, ‘What do I need to do now?’ and ‘What exactly will be uploaded?’ They were also largely unaware of the opt-out regulation.*

[GP13_male_55]

Authentication and registration barriers further constrained patients’ capability and opportunity to engage with the ePA.

*They [patients] simply fail at the registration stage.*

[GP5_male_54]

Individual roles and characteristics shaped how participants evaluated and engaged with the ePA. Within the CFIR role of high-level leaders, active leadership emerged as an important facilitator. Several participants emphasized that implementation required prioritization, coordination, and role modeling by practice leadership. Professional self-concept influenced whether digital work was perceived as an administrative burden or as an opportunity to reduce manual tasks.

*One of my guiding principles is: before we do something ourselves, let the machine do it and have a coffee.*

[GP2_male_48]

Technical affinity and prior experience with digital systems facilitated use. Participants with higher digital confidence described the ePA as easier to approach than colleagues with lower technical affinity. At the same time, digital fatigue and frustration with the introduction of yet another tool were reported, particularly in practices already confronted with multiple digital applications.

*...some people [in the practice] were a bit annoyed that yet another tool was being introduced.*

[MA5_female_55]

Digital reluctance was not described solely as age-related. Some participants expressed a stable preference for analogue or paper-based work practices, even when they were otherwise familiar with digital systems.

*I’m only in my mid-thirties, and I still need a paper printout in my hand to really see and understand what’s written.*

[MA17_female_33]

Readiness for change varied among individuals. Openness and willingness were described as necessary preconditions for engaging with the ePA. Self-efficacy and personal commitment further influenced implementation engagement. Some participants described taking responsibility independently, even without explicit instruction or delegation.

*That was my own initiative. I did record it as working time, but no one approached me and said, ‘Please take a look at this, it’s important.’ I think it’s simply because I’m personally very committed.*

[MA16_female_36]

### Implementation Process

Within the Implementation Process domain, findings primarily reflected the CFIR constructs of planning and doing. Planning was often informal and relied on individual initiative rather than institutionalized implementation pathways. Execution was characterized by fragmented and gradual integration into routine workflows. Participants often described ePA use as something they approached step by step, depending on available time and interest.

*For me, it was more of a gradual process. I eased into it step by step. Whenever I had time or interest, I read up on it.*

[GP12_female_52]

The initial phase was associated with additional work and time requirements, particularly due to data entry, patient discussions, and consent-related processes.

*At first, it takes an enormous amount of time to enter the data and discuss everything with patients. Patients also have to provide written consent. This involves a considerable time investment.*

[PA1_female_28]

Over time, some participants reported habituation effects and growing routinization, even when the ePA was not yet fully integrated into everyday practice. Actual use varied widely across practices, ranging from daily use to near nonuse despite technical availability.

*At this point, we use the ePA every day.*

[GP8_female_42]

### Patterns of Facilitators and Barriers Across CFIR Domains

**Figure 2.**
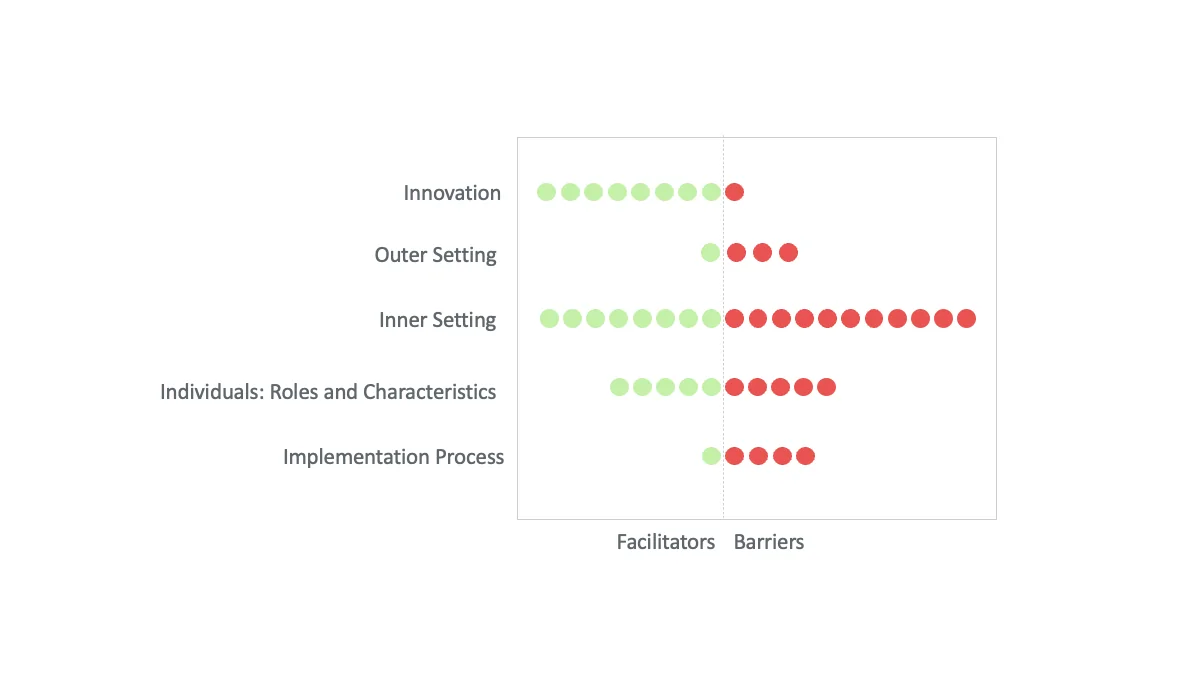
Distribution of facilitator and barrier categories across the Consolidated Framework for Implementation Research domains. Each dot represents one data-driven category presented in Table 2. Green dots indicate facilitators and red dots indicate barriers.

Figure 2 visualizes the distribution of the 47 categories presented in Table 2. Facilitators predominated in the Innovation domain, whereas barriers predominated in the Outer Setting, Inner Setting, and Implementation Process domains. The Individuals domain showed an equal number of facilitators and barriers.

## Discussion

### Principal Findings

This study provides empirical insights into the early implementation phase of the ePA in German outpatient practices from the perspective of HCPs using the CFIR framework. The findings indicate that early implementation was constrained less by professional resistance or perceived system usability than by organizational, technical, and systemic conditions.

The findings are broadly consistent with international evidence on EHR implementation, which has repeatedly identified organizational readiness, leadership engagement, training, workflow integration, technical usability, interoperability, and stakeholder engagement as central implementation determinants [3,21,22]. The specific contribution of this study lies in showing how these established determinants manifest during the early rollout of a nationwide opt-out EHR in German outpatient care, where mandatory availability, heterogeneous PMS functionality, fragmented governance responsibilities, and limited patient activation shaped implementation under routine conditions.

At the organizational level, the findings indicate that local implementation capacity was essential for translating technical availability into routine use. This interpretation is consistent with international evidence highlighting organizational readiness as critical for digital transformation in health care [23-25].

Practices in which leadership actively set priorities and acted as role models were better able to integrate the ePA into routine workflows [26]. However, limited staffing, persistent time constraints, and unstable IT infrastructures restricted the capacity of practices to undertake this implementation work. Implementation frequently occurred under conditions of structural scarcity, intensifying pressure on existing routines and increasing reliance on individual additional effort. This indicates that ePA implementation should be treated as an organizational change process rather than as the installation of an additional technical function. In the absence of sustained investment in human resources, training, and infrastructure, early implementation gains are unlikely to be durable.

The distinction between acceptance of the ePA and the maturity of its surrounding technical infrastructure is particularly important. Although HCPs generally perceived the ePA as intuitive and potentially useful, implementation problems arose when the infrastructure did not support efficient use, a pattern also reported in previous studies [27,28]. Accordingly, complexity arising from technical instability, interface limitations, and additional workflow steps should be interpreted primarily as an indicator of limited system maturity rather than as evidence of fundamental resistance to the ePA [29,30].

The importance attributed to trialability further highlights the value of protected testing environments that allow technical, organizational, and workflow-related adaptation without immediate performance pressure. Evidence from other national EHR implementations similarly indicates that phased rollouts support stabilization and professional engagement during early implementation [31,32]. This supports an iterative, user-centered approach in which technical stabilization and workflow adaptation continue throughout rollout rather than ending with initial deployment. The practice-level experiences also point to a broader governance problem: the usefulness of a national EHR depends on coordinated implementation by regulators, infrastructure providers, software vendors, and organizations across care sectors. The reported variation in PMS functionality and cross-sectoral participation therefore reflects broader difficulties in governing interoperable digital infrastructures in fragmented health care systems and is consistent with international evidence identifying governance deficits and limited enforceability of standards as key barriers to large-scale EHR implementation [4].

The implementation processes were largely informal and incremental, relying on pragmatic adaptation rather than structured planning. The utilization of local “champions” in the implementation of EHRs has been previously documented in other studies [7,33,34], indicating that informal implementation can indeed promote early adoption; however, the absence of formalized support structures can potentially compromise sustainability. Formalized implementation pathways, clearly defined responsibilities, and the sharing of practice-oriented strategies may therefore reduce dependence on individual initiative and support more consistent integration.

Financial reimbursement was perceived as an initial motivator but proved insufficient for sustained integration of the ePA into routine practice. Incentives focused on discrete reimbursable activities primarily addressed short-term workload demands without resolving underlying organizational and workflow-related constraints. Comparable patterns have been reported internationally, where incentives supported formal compliance but had limited impact on deeper workflow integration and intrinsic motivation [29,35].

Incentive structures should therefore support the sustained work of implementation, including training, workflow redesign, and technical support, rather than focusing solely on discrete reimbursable activities. Patient activation should likewise be understood as a system-level implementation condition rather than as the responsibility of individual practices. From the perspective of HCPs, limited information and support outside practices shifted counseling and technical assistance into routine consultations without corresponding resources. Coordinated communication, accessible authentication support, and clearer responsibilities among health insurers and other system actors are therefore necessary to enable active patient participation.

The findings indicate that early ePA implementation was predominantly driven by a supply-side logic. In the absence of coordinated patient engagement strategies, technical availability alone was insufficient to generate meaningful use. Evidence of this unrealized potential is also reflected in a population-based survey on the ePA. In this survey, 1500 individuals insured under the statutory health system were questioned about their use of the ePA. Only 12% reported having actively set up and used their ePA at least once. In contrast, 79% were classified as passive users who had not independently accessed their records, while 7% reported having opted out of ePA use [36].

Taken together, the evidence suggests that technical implementation does not by itself ensure integration into patients’ everyday lives. A coordinated and comprehensive communication and integration strategy is still largely lacking, meaning that many potential users are not being systematically informed. Instead, the introduction of the ePA appears to follow a predominantly policy-driven technology push logic, prioritizing infrastructure provision over patients’ needs and everyday use practices [37]. Consequently, persistently low usage rates should be interpreted as a structural implementation issue rather than a lack of acceptance among patients. Persistently low active use should therefore be addressed through coordinated patient engagement and implementation support rather than being attributed solely to a lack of acceptance.

The practical implications summarized in Table 3 follow from the interdependence of these determinants. Technical stability and enforceable standards shape workflow compatibility and resource requirements within practices; organizational support influences whether HCPs can adapt to the innovation; and patient information strategies affect both active use and the counseling burden placed on practices. The recommendations therefore focus on coordinated action across CFIR domains rather than on isolated measures (Table 3).

**Table 3.** Consolidated Framework for Implementation Research–based practical implications for *elektronische Patientenakte* (German electronic health record) implementation.

Domain	Key findings (empirical basis)	Design and implementation implications
Intervention Characteristics	Perceived complexity was primarily attributed to technical instability, system disruptions, and additional workflow steps rather than to the ePA concept itself Adaptability depended on feedback processes and visible system improvements Financial incentives triggered initial use but did not ensure sustained integration	Prioritizing technical stability and interoperable system architecture during rollout, before expanding functionality Establishing continuous user-centered refinement through structured feedback loops between practices and vendors Aligning incentive structures with long-term workflow integration rather than discrete reimbursable tasks
Process	Implementation was largely informal, incremental, and driven by individual initiative, with a high workload in the early phase Early implementation was associated with increased time burden and additional tasks	Developing formalized implementation pathways with defined roles and structured, role-based training strategies Introducing phased onboarding and temporary workload mitigation during early rollout phases
Outer Setting	Heterogeneity in PMS^a^ functionality and lack of enforceable standards were perceived as major barriers Lack of cross-sectoral integration limited the perceived usefulness of the ePA Low patient demand and information deficits limited engagement	Implementing enforceable interoperability and performance standards across vendors Promoting cross-sectoral data integration with shared accountability mechanisms Coordinating patient engagement and digital literacy strategies at the system level
Inner Setting	Implementation was constrained by technical infrastructure, including unstable systems and network limitations Access to knowledge depended on individual initiative, with gaps in structured training	Investing in robust digital infrastructure capacity to support routine use Providing structured digital competence development with protected learning time
Individuals: Roles and Characteristics	Technical affinity, self-efficacy, and professional attitudes toward digital work shaped how HCPs^b^ engaged with the ePA, while digital fatigue and preference for paper-based work could hinder uptake	Providing role-sensitive training, peer support, and low-threshold assistance for HCPs with varying levels of digital confidence, while avoiding additional digital burden and supporting positive experiences of use

a PMS: practice management system.

b HCP: health care professionals.

Although the implications are presented according to CFIR domains, they should not be understood as isolated recommendations. The findings indicate important overlap and synergy across domains. For example, technical stability and enforceable vendor standards in the Outer Setting directly affect perceived complexity and relative advantage at the innovation level, while also shaping workflow compatibility and resource use within practices. Similarly, structured training and low-threshold knowledge resources support individual self-efficacy, reduce reliance on informal initiative, and facilitate more consistent implementation processes. Patient information strategies at the system level may also reduce counseling burden within practices and increase the perceived usefulness of the ePA in routine care. Thus, successful implementation requires coordinated action across technical, organizational, professional, and policy levels rather than domain-specific measures alone.

To our knowledge, this is the first study examining the early nationwide rollout of the opt-out ePA in Germany. Its strengths include the use of an established theoretical framework, a rigorous qualitative methodology, and the inclusion of HCPs from different disciplines and regions across Germany, allowing for a broad range of perspectives. However, the findings are based on self-reported experiences and reflect participants’ individual practice contexts, which may limit generalizability. The sample may further be biased toward early adopters and digitally engaged professionals, as prior ePA exposure was an inclusion criterion and participants self-selected into the study after receiving invitations through professional organizations. While prior ePA exposure was necessary to obtain experience-based accounts of early implementation, this criterion may have led to an overrepresentation of participants with higher engagement, more positive attitudes, or greater implementation readiness. Recruitment was supported by selected regional Associations of Statutory Health Insurance Physicians and a professional organization, which may have contributed to regional concentration and may limit the representativeness of the sample for all outpatient practices in Germany. Nurse practitioners were not included because the study focused on the German outpatient general practice setting, where nurse practitioner roles are not routinely established as part of standard care teams. In addition, the cross-sectional design captures implementation at an early stage and does not allow for conclusions about long-term use or sustainability. Patient perspectives were not included, limiting insights into user-side barriers and facilitators. The study also focused on outpatient general practice and therefore does not capture implementation experiences in other care sectors, such as hospitals, specialist care, pharmacies, or rehabilitation settings. This is relevant because the perceived usefulness of the ePA depends strongly on participation and information exchange across sectors. Patient perspectives are being investigated in a separate component of the broader ePA4all project and will be reported [15].

### Conclusion

The early implementation of the ePA in German outpatient care is characterized less by professional resistance or acceptance than by organizational, technical, and systemic constraints. Although the ePA is generally perceived as useful and intuitive under stable conditions, its routine use still depends heavily on contextual factors. Technological infrastructure alone is not sufficient for meaningful digital transformation in health care. Future strategies should therefore pursue a coordinated, multistage approach that combines technical development with organizational support, regulatory control, and patient empowerment to enable long-term, routine use of the ePA.

## Supplementary material

10.2196/94620 Multimedia Appendix 1 Interview guide.

10.2196/94620 Multimedia Appendix 2 Coding tree.

10.2196/94620 Checklist 1 COREQ checklist.

## Abbreviations

CFIR: Consolidated Framework for Implementation Research
COREQ: Consolidated Criteria for Reporting Qualitative Research
EHR: electronic health record
ePA: *elektronische Patientenakte* (German electronic health record)
HCP: health care professionals
KVB: Association of Statutory Health Insurance Physicians Berlin
KVN: Association of Statutory Health Insurance Physicians Lower Saxony
KVSH: Association of Statutory Health Insurance Physicians Schleswig-Holstein
KVWL: Association of Statutory Health Insurance Physicians Westphalia-Lippe
PMS: practice management system
