# Supplementary material for: Early Implementation of a Nationwide Opt-Out Electronic Health Record for Outpatient Care in Germany: Qualitative Semistructured Interview Study
Source: J Med Internet Res. 2026 Sep 21;28:e94620. doi: 10.2196/94620 (PMC13592561; doi:10.2196/94620)
Supplement: Supplementary file 1 [file jmir-v28-e94620-s001.pdf]

Interview guide

Main Question / Narrative Prompt	*Check aspects*	*Probing Prompts*	*Follow-up Questions*
When you think about the introduction of the ePA in your organization – how did you experience the implementation?	**PROCESS**<br><br>**Planning:** Was there a structured implementation plan for the ePA?<br>**Stakeholder involvement:** Who was involved? Were users involved at an early stage?<br>**Communication strategy:** How was internal communication handled?<br>**Reflection & evaluation:** Were there feedback loops, adjustments, or exchange formats?<br>**Sustainability:** Measures to ensure continued use	How did the implementation of the ePA proceed in your organization?<br><br>Who was primarily involved in the implementation?<br><br>Which strategies or measures were particularly helpful?<br><br>What would you do differently next time, or what would you wish for?	Could you elaborate on that?<br><br>What was that like for you?<br><br>How do you see it?<br><br>Could you go into more detail?<br><br>Could you give an example?<br><br>What do you mean exactly?
How would you assess the usability of the ePA?	**INTERVENTION CHARACTERISTICS**<br><br>**Complexity:** How complex is the ePA perceived to be in use?<br>**Compatibility:** How well does the ePA fit with existing workflows, routines, and systems?<br>**Adaptability:** Can the ePA be flexibly adapted to local needs?<br>**Perceived benefit:** What advantages does the ePA have compared to previous solutions?<br>**Resistance:** including cultural-	To what extent is the ePA compatible with existing processes from your perspective?<br><br>What level of effort did you experience during the implementation?<br><br>How well does the ePA fit your existing workflows and digital systems?<br><br>What advantages do you see in using the ePA compared to previous procedures?	

	professional resistance / loss of autonomy / discrepancy between actual care practice and documentation practice<br>**Quality and design:** Technical reliability, user interface, usability<br>**Cost-benefit assessment:** Time, money, effort; in relation to expected benefits	How do you assess the technical stability and user interface of the ePA? How would you rate its usability?<br><br>In your view, is the effort required for implementing and using the ePA proportionate to its benefits?	
Which external framework conditions (e.g., legal requirements, data protection, patient access to the ePA) influence your use of the ePA?	**OUTER SETTING**<br><br>**Patient needs and pressure:** Do patients have expectations regarding the ePA?<br>**External policies and incentives:** Influence of legal requirements, deadlines, sanction/reward mechanisms (e.g., via statutory health insurance)<br>**Networking with other institutions:** Need for cross-sector communication<br>**External support:** Role of associations, industry partners, Associations of Statutory Health Insurance Physicians, gematik, etc.	To what extent do patients have expectations or demands regarding the ePA?<br><br>Have patients actively approached you about the ePA or asked you to use it?<br><br>How do legal requirements, deadlines, or incentives influence your work with the ePA?<br><br>What impact do expectations from professional associations or payers have?<br><br>What role do external partners such as KVen or gematik play in the implementation?<br><br>Are there needs or challenges in communicating with other institutions (e.g., specialists) via the ePA?	

How or by whom is your organization supported in implementing the ePA?	**INNER SETTING**<br>**Organizational structure:** Size, IT infrastructure, availability of resources<br>**Communication channels:** Is there transparent communication about the ePA within the team/organization?<br>**Learning culture:** Openness to new technologies, error culture<br>**Leadership and support:** Do leaders actively support implementation?<br>**Availability of time/personnel/training:** Are training, support, and time buffers available?	Are there clear responsibilities, resources, or training opportunities?<br><br>What role do the technical and personnel resources of your organization play in the introduction of the ePA?<br><br>How would you describe the communication and learning culture regarding digitalization in your organization?	
How would you assess your own competencies and attitudes toward digital applications?	**CHARACTERISTICS OF INDIVIDUALS**<br><br>**Knowledge and understanding of the ePA:** Awareness of functions, purpose, and potential<br>**Self-efficacy:** Confidence in using the ePA<br>**Attitudes toward digitalization:** Openness or skepticism<br>**Individual experiences:** Previous experiences with digital solutions<br>**Values and motivation:** Professional ethos, understanding of data protection, willingness to innovate	How well informed do you feel about the functionality and goals of the ePA?<br><br>How confident do you feel in using the ePA?<br><br>What do you do when something does not work as expected when using the ePA?<br><br>How do your colleagues handle the ePA?<br><br>What experiences, beliefs, or concerns do you encounter in dealing with the ePA?	
