# Supplementary material for: Early Implementation of a Nationwide Opt-Out Electronic Health Record for Outpatient Care in Germany: Qualitative Semistructured Interview Study
Source: J Med Internet Res. 2026 Sep 21;28:e94620. doi: 10.2196/94620 (PMC13592561; doi:10.2196/94620)
Supplement: Supplementary file 2 [file jmir-v28-e94620-s002.pdf]

Multimedia Apendix 3: Coding tree

Innovation	Complexity	Perceived intuitive usability	*“It’s really, really completely straightforward. It’s genuinely simple. It’s literally one click and the ePA opens immediately.”* (ePA_M3_Ä16_male_49)
	Complexity	Increased perceived complexity resulting from technical disruptions or system instability	*“Sometimes it doesn’t work, which still happens occasionally. Then you’re sitting there and don’t know why, and it ends up taking five minutes.”* (ePA_M3_Ä12_female_52)
	Trialability	Protected trial phase without any obligation	*“This kind of testing phase was really invaluable and absolutely essential. It’s something that should definitely be done in the future as well.”*(ePA_M3_Ä5_male_54)
	Trialability	Use of the trial phase to optimize clinical processes and workflows	*“Before it became mandatory, it was important to first try out the workflow in practice, in order to identify problems early on and discuss and resolve them with our IT service provider.”* (ePA_M3_Ä14_male_61)
	Adaptability	Visible system modifications and refinements based on user feedback	*“After a few weeks or months, you can actually see that your feedback has been heard, for example, when a button is no longer in the top right corner but has been moved to the bottom left, where it is easier to reach.”* (ePA_M3_Ä5_male_54)
	Relative Advantage	Increasing benefits with growing usage	*“For us, the ePA is genuinely useful, very useful, and it becomes more useful every day. It’s not hindering my work in any way.”* (ePA_M3_Ä5_male_54)
	Relative Advantage	Reduction of redundant examinations	*“...that at some point this will no longer be necessary, because every specialist takes their own blood samples anyway. So that such redundant examinations can simply be avoided.”* (ePA_M3_Ä8_female_42)
	Relative Advantage	Improved availability of information in routine clinical practice	*“The patient has everything with them, because they often don’t bring their medication plan or their reports, you know.”* (ePA_M3_MFA4_female_33)
	Relative Advantage	Increased transparency and enhanced patient engagement	*“Patients also have a much better overview of their own health and of what has actually been done."* (ePA_M3_PA2_female_27)
Outer Setting	Financing	Financial incentives as an initial trigger, with limited effect on sustained integration	*“At the moment, the main excitement is really about rushing to claim the initial data entry fee before it gets abolished again (laughs). Many colleagues just want to collect the eleven euros.”* (ePA_M3_Ä14_male_61)
	Policies and laws	Perceived need for stronger commitment, accountability, and enforcement toward PMS manufacturers	*“There simply need to be binding obligations for the PMS software providers. What’s missing is accountability and sanctions.”* (ePA_M3_Ä2_male_48)
	Partnerships and Connections	Sectoral implementation gaps	*“I do this with my patients as well. I adapt things [in the ePA] when I change something myself. But it’s all pointless if they end up in hospital and there’s no one there who can even open an electronic patient record.”* (ePA_M3_Ä13_male_55)
	Partnerships and Connections	Insufficient information provided to patients by health insurance companies	*“Health insurers need to inform patients properly.”* (ePA_M3_Ä14_male_61)
	Patient Needs and Resources	Counseling and support efforts fall to medical practices but are perceived as outside their responsibility or as overly burdensome	*“We are not a counseling service. That goes beyond our remit.”* (ePA_M3_MFA18_female_56)
Inner Setting	Access to Knowledge and Information	Structured, low-threshold knowledge resources provided by PMS manufacturers	*“A proper e-learning platform (...) with numerous short, easy-to-understand video clips.”* (ePA_M3_Ä5_male_54)

	Access to Knowledge and Information	Insufficient, non-practice-oriented, or absent training opportunities	*"I would say that what is missing in our setting is proper training. Basically an introduction."* (ePA_M3_PA2_female_27)
	Access to Knowledge and Information	Uncertainty regarding legal issues and handling of sensitive data	*"I don't deny that the patient record can make sense in general. But for people with stigmatizing conditions, who don't want others to know that they take HIV medication or opioids for addiction, it is communicated far too little how they can prevent this."* (ePA_M3_Ä3_male_58)
	Structural Characteristics – Information Technology Infrastructure	Technical infrastructure limitations as a burden	*"I believe that the IT infrastructure, the overall setup, still needs to be expanded considerably in order to enable low-barrier use."* (ePA_M3_PA3_female_31)
	Available Resources	Limited staffing resources and persistent time constraints	*"We simply don't have the time to think about uploading data to the ePA, because there are already four other patients waiting."* (ePA_M3_PA2_female_27)
	Available Resources	High investment costs and technical dependencies	*"When you take all of this into account, the workload is enormous and costly."* (ePA_M3_MFA4_female_33)
	Leadership Engagement	Active leadership engagement	"And of course, the initiative has to come from the practice leader; otherwise, it simply won't work." (ePA_M3_Ä2_male_48)
	Culture – Learning-Centeredness	Learning by doing	*"After that, everything was basically over, and it became a matter of learning by doing."* (ePA_M3_Ä5_male_54)
	Culture – Learning-Centeredness	Established formats for internal exchange and reflection	*"We have a quality circle in our practice, where there is a lot of exchange."* (ePA_M3_Ä12_female_52)
	Compatibility	Workflow-neutral integration into existing routines	*"It hasn't really changed our way of working."* (ePA_M3_Ä5_male_54)
	Compatibility	Media discontinuities caused by parallel documentation systems	*"Then I would have to download it each time, save it, and transfer it into [name of PMS]. Of course, we can't manage that."* (ePA_M3_MFA4_female_33)
	Compatibility	Dependence of compatibility on the respective PMS	*"A friend of mine uses a different system, and for them it works relatively smoothly. They can upload documents easily and even upload several at once. We always have to upload them individually."* (ePA_M3_Ä14_male_61)
	Compatibility	Incompatible data formats and complex conversion processes	*"In theory, we first document them in our system and then have to convert them into a Word file, print that as a PDF, convert it into PDF/A within the PMS, and only then upload it."* (ePA_M3_Ä14_male_61)
	Compatibility	Negative interactions with other digital applications	*"Since the ePA was introduced, electronic prescribing has been working less reliably."* (ePA_M3_MFA18_female_56)
	Communications	Continuous internal communication and team feedback as implementation facilitators	*"Bringing the team on board and, above all, receiving feedback from the team, about where the problems are."* (ePA_M3_Ä5_male_54)
	Structural Characteristics – Work Infrastructure	Structurally embedded roles for digital implementation	*"We have a digital manager and she works very closely with the team, closer than I do as the practice lead (...) and together with her, we regularly engage with the team."* (ePA_M3_Ä5_male_54)
	Structural Characteristics – Work Infrastructure	Restricted structural flexibility in larger organizations	*"We simply don't have the capacity to employ someone like that. We are light-years away from being able to do so."* (ePA_M3_PA2_female_27)
	Tension for Change	Collectively perceived necessity for change within the organization	*"We are all in the same boat. And if one person doesn't row, we all have a problem."* (ePA_M3_Ä2_male_48)
	Culture	Positively framed, digitally oriented practice culture	*"We work completely digitally, and the nursing staff are fully on board. We try to implement this wherever possible and also contribute ideas on how processes could be further digitalized."* (ePA_M3_Ä8_female_42)

Individuals: Roles and Characteristics	Innovation Recipients – Motivation	HCP-perceived low patient demand and limited perceived relevance of the ePA	*"The ePA hardly ever comes up in consultations with patients. Patients ask about it very rarely."* (ePA_M3_Ä5_male_54)
	Innovation Recipients – Capability	HCP-perceived knowledge deficits among patients	*"Patients often asked, 'What do I need to do now?' and 'What exactly will be uploaded?' They were also largely unaware of the opt-out regulation."* (ePA_M3_Ä13_male_55)
	Innovation Recipients – Opportunity	Authentication and registration barriers affecting patients' access to the ePA	*"They [patients] simply fail at the registration stage."* (ePA_M3_Ä5_male_54)
	High-Level Leaders – Motivation	Professional self-concept and appraisal of digital work	*"One of my guiding principles is: before we do something ourselves, let the machine do it and have a coffee."* (ePA_M3_Ä2_male_48)
	Innovation Deliverers – Motivation	Individual level of technical affinity and prior experience with digital systems	*"Of course, it comes more easily to me than to some others who are not very technically inclined."* (ePA_M3_MFA4_female_33)
	Innovation Deliverers – Capability	Digital fatigue and frustration related to the introduction of new tools	*"…some people [in the practice] were a bit annoyed that yet another tool was being introduced."* (ePA_M3_MFA5_female_55)
	Innovation Deliverers – Motivation	Preference for analogue or paper-based work practices	*"I'm only in my mid-thirties, and I still need a paper printout in my hand to really see and understand what's written."* (ePA_M3_MFA17_female_33)
	Innovation Deliverers – Motivation	Individual readiness for change and personal initiative	*"Well, you also have to be open to it, right? You have to actually want it."* (ePA_M3_MFA5_female_55)
	Innovation Deliverers – Motivation	Self-efficacy and personal commitment as individual-level determinants of implementation engagement	*"That was my own initiative. I did record it as working time, but no one approached me and said, 'Please take a look at this, it's important.' I think it's simply because I'm personally very committed."* (ePA_M3_MFA16_female_36)
Implementation Process	Planning	Informal planning with reliance on individual initiative	*"You really have to take the initiative yourself."* (ePA_M3_Ä2_male_48)
	Doing	Fragmented integration into routine workflows	*"For me, it was more of a gradual process. I eased into it step by step. Whenever I had time or interest, I read up on it."* (ePA_M3_Ä12_female_52)
	Doing	Additional work and extra time required in the initial phase	*"At first, it takes an enormous amount of time to enter the data and discuss everything with patients. Patients also have to provide written consent. This involves a considerable time investment."* (ePA_M3_PA1_female_28)
	Adapting	Habituation effects and growing routinization over time	*"It is not yet fully integrated in general. However, it is available, and we are getting used to it."* (ePA_M3_MFA20_female_42)
	Doing	Wide range of actual uses in everyday practice	*"At this point, we use the ePA every day." / "So far, I haven't experienced anyone in our practice actually using it."* (ePA_M3_Ä8_female_42; ePA_M3_PA2_female_27)

Note: Participant identifiers indicate the study identifier, participant group, participant number, sex, and age. "Ä" refers to general practitioners (Ärztinnen/Ärzte), "MFA" to medical assistants (Medizinische Fachangestellte), and "PA" to physician assistants. For example, ePA_M3_Ä16_male_49 refers to general practitioner 16, male, aged 49 years.)
